# Long-term air pollution exposure and self-reported morbidity: A longitudinal analysis from the Thai cohort study (TCS)

**DOI:** 10.1016/j.envres.2020.110330

**Published:** 2021-01

**Authors:** Kanawat Paoin, Kayo Ueda, Thammasin Ingviya, Suhaimee Buya, Arthit Phosri, Xerxes Tesoro Seposo, Sam-ang Seubsman, Matthew Kelly, Adrian Sleigh, Akiko Honda, Hirohisa Takano, Thai Cohort Study Team Thailand, Jaruwan Chokhanapitak, Chaiyun Churewong, Suttanit Hounthasarn, Suwanee Khamman, Daoruang Pandee, Suttinan Pangsap, Tippawan Prapamontol, Janya Puengson, Wimalin Rimpeekool, Yodyiam Sangrattanakul, Sam-ang Seubsman, Boonchai Somboonsook, Nintita Sripaiboonkij, Pathumvadee Somsamai, Benjawan Tawatsupa, Arunrat Tangmunkongvorakul, Duangkae Vilainerun, Wanee Wimonwattanaphan, Thai Cohort Study Team Australia, Chris Bain, Emily Banks, Cathy Banwell, Janneke Berecki-Gisolf, Bruce Caldwell, Gordon Carmichael, Tarie Dellora, Jane Dixon, Sharon Friel, David Harley, Susan Jordan, Matthew Kelly, Tord Kjellstrom, Lynette Lim, Roderick McClure, Anthony McMichael, Tanya Mark, Adrian Sleigh, Lyndall Strazdins, Tam Tran, Vasoontara Yiengprugsawan, Jiaying Zhao

**Affiliations:** a Department of Environmental Engineering, Graduate School of Engineering, Kyoto University, Kyoto, Japan; b Graduate School of Global Environmental Sciences, Kyoto University, Kyoto, Japan; c Department of Family and Preventive Medicine, Faculty of Medicine, Prince of Songkla University, Songkhla, Thailand; d Medical Data Center for Research and Innovation, Faculty of Medicine, Prince of Songkla University, Songkhla, Thailand; e Department of Environmental Health Sciences, Faculty of Public Health, Mahidol University, Bangkok, Thailand; f School of Tropical Medicine and Global Health, Nagasaki University, Nagasaki, Japan; g School of Human Ecology, Sukhothai Thammathirat Open University, Nonthaburi, Thailand; h Department of Global Health, Research School of Population Health, Australian National University, Canberra, Australia

**Keywords:** Long-term air pollution exposure, High blood pressure, High blood cholesterol, Diabetes, Cardiovascular disease risk factors

## Abstract

**Background:**

Several studies have shown the health effects of air pollutants, especially in China, North American and Western European countries. But longitudinal cohort studies focused on health effects of long-term air pollution exposure are still limited in Southeast Asian countries where sources of air pollution, weather conditions, and demographic characteristics are different. The present study examined the association between long-term exposure to air pollution and self-reported morbidities in participants of the Thai cohort study (TCS) in Bangkok metropolitan region (BMR), Thailand.

**Methods:**

This longitudinal cohort study was conducted for 9 years from 2005 to 2013. Self-reported morbidities in this study included high blood pressure, high blood cholesterol, and diabetes. Air pollution data were obtained from the Thai government Pollution Control Department (PCD). Particles with diameters ≤10 μm (PM_10_), sulfur dioxide (SO_2_), nitrogen dioxide (NO_2_), ozone (O_3_), and carbon monoxide (CO) exposures were estimated with ordinary kriging method using 22 background and 7 traffic monitoring stations in BMR during 2005–2013. Long-term exposure periods to air pollution for each subject was averaged as the same period of person-time. Cox proportional hazards models were used to examine the association between long-term air pollution exposure with self-reported high blood pressure, high blood cholesterol, diabetes. Results of self-reported morbidity were presented as hazard ratios (HRs) per interquartile range (IQR) increase in PM_10_, O_3_, NO_2_, SO_2_, and CO.

**Results:**

After controlling for potential confounders, we found that an IQR increase in PM_10_ was significantly associated with self-reported high blood pressure (HR = 1.13, 95% CI: 1.04, 1.23) and high blood cholesterol (HR = 1.07, 95%CI: 1.02, 1.12), but not with diabetes (HR = 1.05, 95%CI: 0.91, 1.21). SO_2_ was also positively associated with self-reported high blood pressure (HR = 1.22, 95%CI: 1.08, 1.38), high blood cholesterol (HR = 1.20, 95%CI: 1.11, 1.30), and diabetes (HR = 1.21, 95%CI: 0.92, 1.60). Moreover, we observed a positive association between CO and self-reported high blood pressure (HR = 1.07, 95%CI: 1.00, 1.15), but not for other diseases. However, self-reported morbidities were not associated with O_3_ and NO_2_.

**Conclusions:**

Long-term exposure to air pollution, especially for PM_10_ and SO_2_ was associated with self-reported high blood pressure, high blood cholesterol, and diabetes in subjects of TCS. Our study supports that exposure to air pollution increases cardiovascular disease risk factors for younger population.

## Highlights

• Evidence on health effects of long-term air pollution exposure are still limited in Southeast Asia.
• SO_2_ was associated with an increased risks of self-reported high blood pressure, high blood cholesterol, and diabetes.
• Increase of PM_10_ was also associated with the incidences of high blood pressure and high blood cholesterol.
• The effects were observed even at the level below the National Standard Limit.
• This study suggests air pollution increases cardiovascular disease risk factors for younger population.

## 1 Introduction

Air pollution is one of the most serious environmental problems worldwide, especially in developing countries. Exposure to air pollution has detrimental effects on health and is one of the hardest environmental risks to avoid. Many epidemiological studies have found the effects of air pollution on mortality and morbidity for cardiovascular (Brook et al., 2010; Gold and Mittleman, 2013; Pope et al., 2004) and respiratory diseases (Analitis et al., 2006; Dockery and Pope, 1994; Hoek et al., 2013; Middleton et al., 2008). Additionally, long-term exposure to air pollution has been linked with hypertension (Bai et al., 2018; Coogan et al., 2012, 2017; Zhang et al., 2018), diabetes (Andersen et al., 2012; Brook et al., 2013; Eze et al., 2014; Hansen et al., 2016; Liang et al., 2019; Park et al., 2015; Qiu et al., 2018), dyslipidaemias (Bo et al., 2019a; Mao et al., 2020; Yang et al., 2018), liver cancer (Pan et al., 2016; Pedersen et al., 2017), and kidney disease (Bowe et al., 2017a, 2017b).

As with other developing countries, Thailand experiences air pollution, mostly from vehicular emissions in cities, biomass burning and transboundary haze in rural and border areas, and industrial discharges in concentrated industrialized zones (Vichit-Vadakan and Vajanapoom, 2011). These processes emit air pollution in the atmosphere and seriously affect human and environmental health. According to the Pollution Control Department (2019), the annual PM_10_ (a particulate matter less than 10 μm in aerodynamic diameter), PM_2.5_ (a particulate matter less than 2.5 μm in aerodynamic diameter), and ozone (O_3_) had still exceeded their national standards in many areas of Thailand, whereas other gaseous pollutants such as nitrogen dioxide (NO_2_), sulfur dioxide (SO_2_), and carbon monoxide (CO) were well below that standard. However, the concentrations of NO_2_, SO_2_, and CO in some areas of Thailand, especially for Bangkok were periodically above the annual or 24-h mean values as determined by WHO air quality guidelines (WHO Regional Office for Europe, 2006).

Bangkok is the capital and most populous city of Thailand. Over 10 million people (15% of the Thai population) live in Bangkok. The climate in Bangkok is hot (normally above 30 °C) and humid (monthly average range of humidity is between 74% and 85%) throughout the year (Bangkok Climate, 2016). Bangkok has experienced serious urban air pollution problems because of rapid economic development and urbanization. Chuersuwan et al. (2008) has demonstrated that the major sources of PM_10_ at roadside location in Bangkok are vehicle emissions and biomass burning, which contributes roughly 33% each. Other gaseous pollutants, including NO_2_, SO_2_, and CO are also generated from vehicle exhaust. O_3_ in Bangkok could be formed due to vehicle emissions, where ozone precursors have been emitted (i.e. nitrogen oxide (NOx), CO, and volatile organic compounds (VOCs)), and sunlight is present. The favorable meteorological conditions, such as high temperature and strong solar radiation, could also enhance the O_3_ formation in Bangkok. Air pollution's contribution to mortality was greater in Bangkok, than in Hong Kong, Shanghai and Wuhan in China (Taneepanichskul et al., 2018; Wong et al., 2008). Short-term studies in Thailand also found associations between air pollution and respiratory and cardiovascular mortality (Ostro et al., 1999; Taneepanichskul et al., 2018) and morbidity (Buadong et al., 2009; Phosri et al., 2019; Trang and Tripathi, 2014).

Although health effects of air pollutants are well documented in many countries especially in China, North American and Western Europe, long-term studies are still limited in Southeast Asian countries, including Thailand where sources of air pollution, weather conditions, demographic characteristics (Phosri et al., 2019) and lifestyle (Strak et al., 2017) in each country are different. Moreover, no longitudinal cohort study has examined the association between long-term exposure to air pollution and health effects in Thailand. This study examined the association between long-term exposure to air pollution and self-reported morbidities in Bangkok metropolitan region (BMR), Thailand from 2005 to 2013.

## 2 Methods

### 2.1 Study design and participants

The population and recruitment of the Thai Cohort Study (TCS) was previously described in detail (Seubsman et al., 2011, 2012; Sleigh et al., 2008). Briefly, the Thai Health-Risk Transition Project began in 2004 with the aim of studying changes in the health status of the Thai population associated with rapid modernization and industrialization. Part of this study project has involved assembling a cohort of adult community dwelling Thais whose health status could be followed through time along with their risk behavior and socio-demographic and economic profiles. The target population was persons studying by correspondence via Sukhothai Thammathirat Open University (STOU). This group was chosen because STOU students lived throughout the country and display considerable variation in lifestyle, family structure, socio-economic status, domestic and occupational environment and personal behavior.

The cohort population is similar to the general Thai population in terms of median age, geographic residence and median income (Sleigh et al., 2008). They are however, overall, more highly educated than the Thai population. This means this study population is able to represent potential future health transitions in Thailand, as education levels in the general population increase. In 2005 a health questionnaire was mailed out to all students currently enrolled at STOU; 87,151 participants responded and formed the baseline cohort population. Two follow-ups were conducted in 2008/2009 (n = 60,569) and 2012/2013 (n = 42,785). Our study period was from 2005, the start of the cohort, until 2013 when the last survey was completed.

**Fig. 1:**
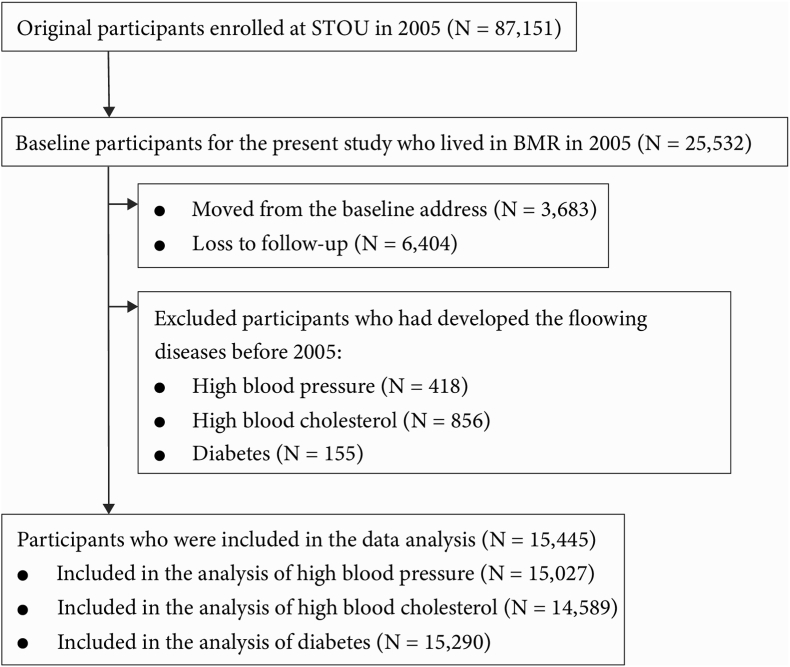
Flowchart of participant selection.

As shown in Fig. 1, we extracted the data of 25,532 subjects in TCS who lived in BMR, including Bangkok, Nonthaburi, Samutprakarn, Samut sakhon, Nakhon pathom, and Pathum thani in 2005. We excluded 3,683 subjects who moved from their baseline address and 6,404 subjects who were lost to follow-up. In addition, those who had developed diabetes (n = 155), high blood pressure (n = 418), and high cholesterol (n = 856) before 2005 were also excluded.

### 2.2 Data collection

Self-reported morbidities, including diabetes (Papier et al., 2017), high blood pressure (Rimpeekool et al., 2017; Thawornchaisit et al., 2014), high cholesterol (Lim et al., 2012; Rimpeekool et al., 2017), and information on other self-reported comorbidities, sex, age, and various subjects associated with health, including demography, social networking, work, health services, injury, environment, food and physical activity, smoking, alcohol and transport collected at 3 time points in 2005, 2009, and 2013 were obtained from TCS participants in the Thai Health Research Project (Seubsman et al., 2011, 2012; Sleigh et al., 2008).

### 2.3 Exposure assessment

Hourly concentrations of PM_10_ and other gaseous pollutants, including SO_2_, NO_2_, CO, and O_3_ were obtained from the Pollution Control Department (PCD) in Thailand. The chemiluminescence method was applied for measuring O_3_ and NO_2_; UV-fluorescence for SO_2_; Tapered Element Oscillation Microbalance (TEOM) for PM_10_; and Non-Dispersive Infrared Detection for CO. Same period daily average temperature (in degrees Celsius; °C) and relative humidity (in percent; %) were obtained from the Thai Meteorological Department. Meteorological data was used from only one meteorological station which is located in Bangkok and had complete data of temperature and relative humidity from 2004 until 2013. Additionally, almost all of study subjects lived in Bangkok. Therefore, we selected the main meteorological station in Bangkok to represent environmental conditions for all participants.

**Fig. 2:**
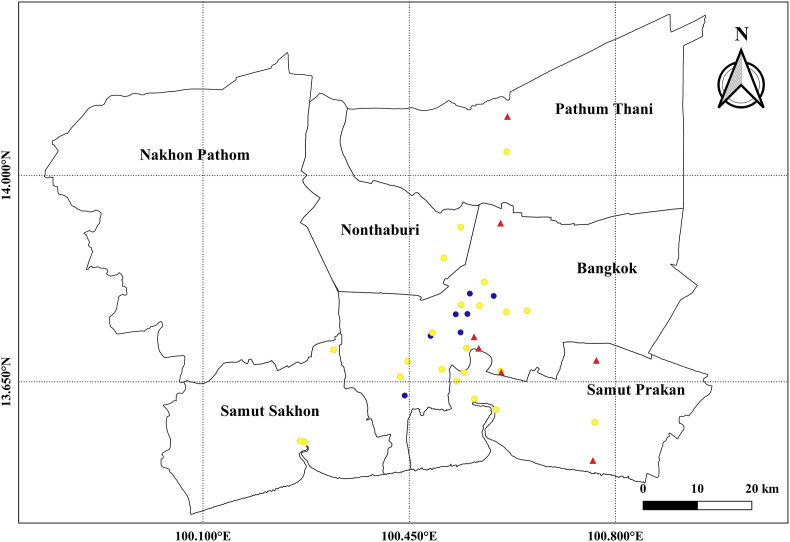
Map of the study area (BMR). Ambient monitoring stations are denoted by yellow circles; traffic monitoring stations are denoted by blue circles; meteorological monitoring stations are denoted by triangles.

**Fig. 3:**
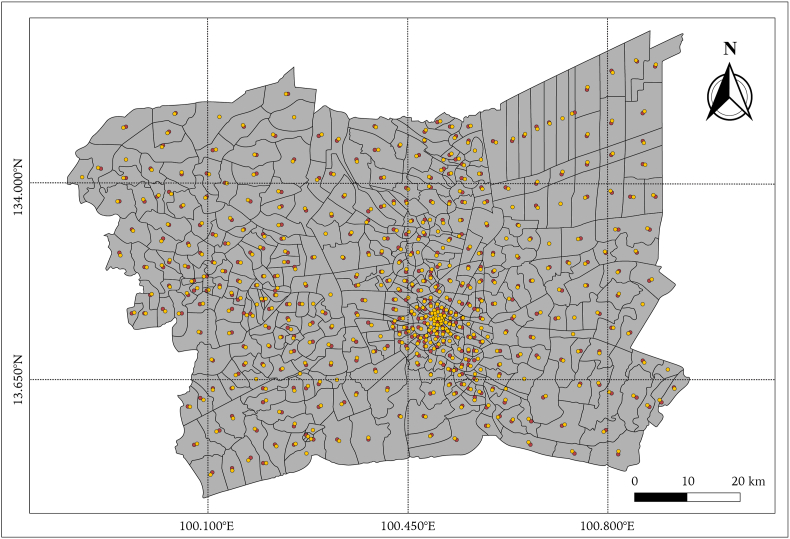
The grid of nearest centroid point of each district in the study area (BMR).

Air pollution exposure, including daily average of PM_10_, SO_2_, NO_2_, CO, and daily maximum 8-hr O_3_ average (Lim et al., 2018) were estimated with ordinary kriging method (Geniaux et al., 2017; Leem et al., 2006; Liu and Rossini, 1996) which is a linear prediction model to estimate a value at a point of an unobserved location for which a variogram is known, based on the weighted average of surrounding monitoring stations (Wackernagel, 1995). We included hourly mean air pollution concentrations as a dependent variable and geographical data, and latitudinal and longitudinal coordinates, as potential predictors (independent variables). We conducted measurements of concentrations of air pollutants from 22 background and 7 traffic monitoring sites in BMR during 2005–2013 (Fig. 2) in order to generate a long-term annual average from all discontinuous site-specific measurements. For the prediction, we generated the grids of 100x100 m. Then the average concentration for each air pollutant at the district levels were estimated by the concentrations from the grids closest to the centroid of each district (Fig. 3). We developed and validated a separate model for each air pollutant to estimate grid-specific air pollution concentrations. Good model performance from 2005 to 2013 was achieved, with leave-one-out cross validation R^2^ value of 0.99 for PM_10_, O_3_, NO_2_, SO_2_, and 0.98 for CO. Model predictions had little bias, with cross-validated slopes (predicted vs observed) of 0.99 for PM_10_, O_3_, NO_2_, SO_2_, and 0.98 for CO.

Air pollution exposure for each subject was averaged from the start of cohort (year 2005) to the year of disease occurrence; the exposure until the end of cohort (year 2013) was considered if the subject did not develop any disease during the cohort period. Moreover, we averaged the exposure until the first follow-up of cohort (year 2009) if the subject without disease in 2009 was lost to follow-up or changed the address in 2013.

### 2.4 Covariates

Information on a wide range of potential covariates was collected using a standard self-administered questionnaire. We used data in 2005 on age (years), sex (male/female), BMI (kg/m^2^), smoking status (never-smokers/former-smokers/current-smokers), smoking intensity (number per day), alcohol drinking (non-users/former-users/occasional-users/current-users), daily alcohol intake (less than 2 glasses/2–3 glasses/4–5 glasses/6 glasses or more), strenuous and moderate exercise (times per week), education level (junior high school or equivalent/completed high school or equivalent/post-high school diploma or certificate/bachelor or higher university degree), average monthly income (≤3,000 Thai baht/3,001–7,000 Thai baht/7,001–10,000 Thai baht/10,001–20,000 Thai baht/20,001–30,000 Thai baht/≥30,000 Thai baht), marital status (single/living with partner or married/divorced or separated/widowed), high fat, sodium and sugar consumption and sugar-sweetened soft drink consumption (never or less than once a month/1–3 times per month/1–2 times per week/3–6 times per week/once a day or more), vegetables and fruit consumption (serves/day), and other self-reported comorbidities (i.e. cancer, chronic bronchitis, asthma, stroke, coronary heart disease).

### 2.5 Statistical analysis

We used a time-varying Cox proportional hazards model to investigate associations between long-term air pollution exposure (PM_10_, O_3_, NO_2_, SO_2_ and CO) and the development of diabetes, high blood pressure, and high cholesterol during 2005–2013. The concentrations of air pollutants were included as time-dependent variables in the Cox regression model. The time-scale used in the Cox regression model is time-in-study (i.e. follow-up time). Person-time was calculated from the enrollment of cohort in 2005 until the year of the occurrence of diabetes or high blood pressure or high cholesterol, loss to follow-up, moving from the study area, death, or end of follow-up, whichever occurred first (Coogan et al., 2012; Liang et al., 2019; Lim et al., 2018). Study subjects were censored at the time of disease occurrence, at the end of the study period (year 2013), or when they were lost to follow-up or move outside the study area.

A wide range of covariates were selected based on the previous literature (Bai et al., 2018; Bo et al., 2019a, 2019b; Lao et al., 2019; Liang et al., 2019; Qiu et al., 2018; Renzi et al., 2018; Zhang et al., 2018). Five models were developed by gradually including these covariates: a crude model (Model I), and a model adjusted for age and sex only (Model II). The 3rd model (Model III) was adjusted for age, sex, body mass index (BMI), smoking status, alcohol intake, physical activity, high fat, sodium and sugar consumption, intake of fruit and vegetables, sugar-sweetened soft drink consumption, marital status, education level, and income. The 4th model (Model IV) was adjusted for covariates in the third model plus 2-year average of temperature and relative humidity during the year of the disease occurrence or the last follow-up and the preceding year were conducted for each air pollutant. Moreover, an additional model (Model V) adjusting for other self-reported comorbidities (i.e. cancer, chronic bronchitis, asthma, stroke, coronary heart disease) and city of residence. Self-reported comorbidities were considered until the start of cohort in 2005.

Two-pollutant models were used to examine the robustness of the effect estimate. The results were presented as hazard ratios (HRs) per interquartile range (IQR) increase in PM_10_, O_3_, NO_2_, SO_2_, and CO, with 95% confidence intervals (CIs). All statistical analyses were conducted using R statistical project (version 3.6.1). P < 0.05 was considered statistically significant.

## 3 Results

As shown in Table 1, we included 15,027, 14,589, and 15,290 subjects for study of high blood pressure, high blood cholesterol, and diabetes, respectively. Around 60% of TCS's subjects were female and their age range was from 17 to 87 years old in 2005. More than 60% of them were younger than 35 years old. More than a half of study subjects lived in Bangkok. Over 80% of study subjects were low to middle-income earners (<20,000 baht/month). In 2005, around 70% of study subjects had a highest attained education level lower than bachelor degree. During this study period (2005–2013), the proportion of study subjects who developed high blood pressure, high cholesterol, and diabetes were around 7.0%, 17.7%, and 2.0%, respectively.

**Table 1:** Basic characteristics of study subjects at the baseline in 2005.

Variables	Entire Population (N = 25,532)	High blood pressure (N = 15,027)	High cholesterol (N = 14,589)	Diabetes (N = 15,290)
Sex, n (%)				
Male	9946 (39.0)	5,671 (37.7)	5,483 (37.6)	5,855 (38.3)
Female	15586 (61.0)	9,356 (62.3)	9,106 (62.4)	9,435 (61.7)

Age, years				
Mean ± SD	30.9 ± 8.4	32.1 ± 8.3	32.0 ± 8.3	32.3 ± 8.5
Range	[16.0, 87.0]	[17.0, 87.0]	[17.0, 87.0]	[17.0, 87.0]

Age groups				
< 35 years old	17950 (70.3)	9,684 (64.4)	9,544 (65.4)	9,743 (63.7)
≥ 35 years old	7581 (29.7)	5,343 (35.6)	5,045 (34.6)	5,547 (35.3)

Body mass index, kg/m^2^				
Mean ± SD	21.8 ± 3.6	21.9 ± 3.5	21.9 ± 3.6	22.0 ± 3.6

Smoking status, n (%)				
Never smokers	18446 (72.2)	11,069 (73.7)	10,746 (73.7)	11,215 (73.3)
Former smokers	4809 (18.8)	2,764 (18.4)	2,687 (18.4)	2,854 (18.7)
Current smokers	2277 (8.9)	1,196 (8.0)	1,158 (7.9)	1,223 (8.0)

Alcohol drinking, n (%)				
Non-users	14828 (58.1)	4,366 (29.1)	4,252 (29.1)	4,438 (29.0)
Former-users	2247 (8.8)	1,301 (8.7)	1,247 (8.5)	1,333 (8.7)
Occasional-users	7015 (27.5)	8,539 (56.8)	8,282 (56.8)	8,660 (56.6)
Current-users	1086 (4.3)	621 (4.1)	604 (4.1)	656 (4.3)

Moderate Exercise, n (%)				
0 session/ week	13089 (51.3)	7,877 (52.4)	7,625 (52.3)	7,995 (52.3)
1-2 sessions/ week	5850 (22.9)	3,409 (22.7)	3,297 (22.6)	3,469 (22.7)
At least three times a week	5461 (21.4)	3,128 (20.8)	3,063 (21.0)	3,193 (20.9)

Education levels, n (%)				
Junior high school or equivalent	1226 (4.8)	705 (4.7)	698 (4.8)	729 (4.8)
Completed high school or equivalent	11730 (45.9)	6,713 (44.7)	6,613 (45.3)	6,821 (44.6)
Post-high school diploma or certificate	6230 (24.4)	3,503 (23.3)	3,434 (23.5)	3,545 (23.2)
Bachelor or higher university degree	6263 (24.5)	4,063 (27.0)	3,805 (26.1)	4,150 (27.1)

Income (monthly), n (%)				
≤ 3,000 Baht	1496 (5.9)	792 (5.3)	789 (5.4)	800 (5.2)
3,001 – 7,000 Baht	6617 (25.9)	3,475 (23.1)	3,473 (23.8)	3,512 (23.0)
7,001 – 10,000 Baht	6457 (25.3)	3,722 (24.8)	3,664 (25.1)	3,749 (24.5)
10,001 – 20,000 Baht	6543 (25.6)	4,161 (27.7)	4,006 (27.5)	4,232 (27.7)
20,001 – 30,000 Baht	2046 (8.0)	1,401 (9.3)	1,294 (8.9)	1,443 (9.4)
≥ 30,000 Baht	1877 (7.4)	1,225 (8.2)	1,106 (7.6)	1,294 (8.5)

Incidence of self-reported morbidities (2005-2013), n (%)				
Hypertension		1,055 (7.0)		
Hyper cholesterol			2,589 (17.7)	
Diabetes				304 (2.0)

Prevalence of self-reported comorbidities (until 2005), n (%)				
Cancer	221 (0.9)	102 (0.7)	94 (0.6)	102 (0.7)
Stroke	57 (0.2)	32 (0.2)	30 (0.2)	39 (0.3)
Coronary heart disease	106 (0.4)	54 (0.4)	51 (0.3)	60 (0.4)
Asthma	1013 (4.0)	568 (3.8)	551 (3.8)	576 (3.8)
Chronic bronchitis	550 (2.2)	327 (2.2)	317 (2.2)	329 (2.2)
High blood pressure	1324 (5.2)	-	740 (5.1)	855 (5.6)
Diabetes	325 (1.3)	180 (1.2)	170 (1.1)	-
High cholesterol	2922 (11.4)	1881 (12.5)	-	1993 (13.0)

Marital status				
Single	15875 (62.2)	7,853 (52.3)	7,686 (52.7)	7,930 (51.9)
Living with partner or married	8511 (33.3)	6,500 (43.2)	6,250 (42.8)	6,672 (43.6)
Divorced or separated	979 (3.8)	567 (3.8)	545 (3.7)	582 (3.8)
Widowed	167 (0.7)	117 (0.8)	114 (0.8)	119 (0.8)

High fat consumption (Deep fried food)				
Never or less than once a month	707 (2.8)	433 (2.9)	420 (2.9)	432 (3.0)
1-3 times per month	3583 (14.0)	2,108 (14.0)	2,039 (14.0)	2,153 (14.8)
1-2 times per week	7568 (29.6)	4,481 (29.8)	4,366 (29.9)	4,586 (31.4)
3-6 times per week	9278 (36.3)	5,409 (36.0)	5,227 (35.8)	5,507 (37.7)
Once a day or more	4158 (16.3)	2,458 (16.4)	2,401 (16.5)	2,486 (17.0)

High sodium consumption (Canned food)				
Never or less than once a month	9063 (35.5)	5,510 (36.7)	5,336 (36.6)	5,624 (36.8)
1-3 times per month	10910 (42.7)	6,446 (42.9)	6,257 (42.9)	6,566 (42.9)
1-2 times per week	3894 (15.3)	2,183 (14.5)	2,130 (14.6)	2,204 (14.4)
3-6 times per week	1184 (4.6)	644 (4.3)	627 (4.3)	648 (4.2)
Once a day or more	237 (0.9)	105 (0.7)	103 (0.7)	104 (0.7)

High sugar consumption (Food/dessert with coconut milk)				
Never or less than once a month	3805 (14.9)	2,224 (14.8)	2,164 (14.8)	2,271 (14.9)
1-3 times per month	8495 (33.3)	5,045 (33.6)	4,917 (33.7)	5,143 (33.6)
1-2 times per week	7504 (29.4)	4,449 (29.6)	4,305 (29.5)	4,518 (29.5)
3-6 times per week	4221 (16.5)	2,458 (16.4)	2,363 (16.2)	2,492 (16.3)
Once a day or more	1312 (5.1)	742 (4.9)	729 (5.0)	754 (4.9)

Sugar-sweetened soft drink consumption				
Never or less than once a month	5979 (23.4)	3,761 (25.0)	3,632 (24.9)	3,810 (24.9)
1-3 times per month	6332 (24.8)	3,816 (25.4)	3,693 (25.3)	3,916 (25.6)
1-2 times per week	5598 (21.9)	3,314 (22.1)	3,205 (22.0)	3,356 (21.9)
3-6 times per week	4718 (18.5)	2,587 (17.2)	2,534 (17.4)	2,628 (17.2)
Once a day or more	2668 (10.4)	1,418 (9.4)	1,393 (9.5)	1,443 (9.4)

Vegetables consumption (serves/day)				
0 serve/day	409 (1.6)	238 (1.6)	234 (1.6)	241 (1.6)
1 serve/day	12574 (49.2)	7,475 (49.7)	7,214 (49.4)	7,608 (49.8)
2 serves/day	6913 (27.1)	4,142 (27.6)	4,041 (27.7)	4,223 (27.6)
3 serves/day	2738 (10.7)	1,587 (10.6)	1,543 (10.6)	1,609 (10.5)
More than 3 serves/day	2200 (8.6)	1,223 (8.1)	1,198 (8.2)	1,242 (8.1)

Fruit consumption (serves/day)				
0 serve/day	758 (3.0)	437 (2.9)	433 (3.0)	449 (2.9)
1 serve/day	8637 (33.8)	5,143 (34.2)	4,954 (34.0)	5,243 (34.3)
2 serves/day	5946 (23.3)	3,548 (23.6)	3,445 (23.6)	3,615 (23.6)
3 serves/day	3770 (14.8)	2,265 (15.1)	2,216 (15.2)	2,307 (15.1)
More than 3 serves/day	5725 (22.4)	3,270 (21.8)	3,179 (21.8)	3,305 (21.6)

City of residence, n (%)				
Bangkok	14854 (58.2)	8,719 (58.0)	8,452 (57.9)	8,912 (58.3)
Nonthaburi	3012 (11.8)	1,751 (11.7)	1,668 (11.4)	1,773 (11.6)
Samut Prakarn	2816 (11.0)	1,688 (11.2)	1,649 (11.3)	1,717 (11.2)
Phathum Thani	2246 (8.8)	1,246 (8.3)	1,209 (8.3)	1,252 (8.2)
Nakhon pathom	1486 (5.8)	937 (6.2)	922 (6.3)	942 (6.2)
Samutsakorn	1118 (4.4)	686 (4.6)	689 (4.7)	694 (4.5)

Annual summary values of the environmental variables were presented with their mean, standard deviation (SD), range, and IQR in Table 2. Average concentration of PM_10_ during study period nearly exceeded annual PM_10_ standard limit in Thailand (PM_10_ < 50 μg/m^3^). Other gaseous pollutants in this study were well below their national standards, but there is still no annual standard limit of O_3_ and CO in Thailand. Annual average temperatures ranged from 28.4 °C to 29.3 °C, while annual mean relative humidity ranged from 69.5% to 75.7% (Table 2). As shown in Table 3, air pollutants were positively correlated with each other (P < 0.05), while they were negatively correlated with temperature and relative humidity (P < 0.05).

**Table 2:** Annual average concentration of environmental variables during the study period.

Environmental Variable	Mean ± SD	Range	IQR
PM_10_ (μg/m^3^)	44.4 ± 14.4	20.5–125.3	15.3
O_3_ (ppb)	30.2 ± 5.1	14.1–41.2	7.0
NO_2_ (ppb)	19.4 ± 4.1	12.3–39.2	4.4
SO_2_ (ppb)	5.7 ± 3.2	1.2–15.8	4.2
CO (ppm)	0.72 ± 0.2	0.24–1.7	0.2
Temperature (∗C)	29.0 ± 0.3	28.4–29.3	0.4
Relative humidity (%)	72.7 ± 2.0	69.5–75.7	3.1

**Table 3:** Correlation coefficient between air pollution and weather variables during the study period.

	PM_10_	O_3_	NO_2_	SO_2_	CO
O_3_	0.29				
NO_2_	0.45	0.34			
SO_2_	0.16	0.04	0.09		
CO	0.3	0.17	0.54	0.13	
Temperature	−0.13	−0.06	−0.27	−0.08	−0.24
Relative humidity	−0.34	−0.45	−0.21	−0.18	−0.08

Table 4 presents the estimated HRs in self-morbidities for an increment of IQR in PM_10_, O_3_, NO_2_, SO_2_ and CO in Model I to Model V. Regardless of the model used, PM_10_ and SO_2_ were significantly associated with incidences of high blood pressure and high cholesterol. Furthermore, we found the positive association between CO exposure and incidence of high blood pressure, but not for high cholesterol and diabetes. In contrast, O_3_ and NO_2_ generally showed negative association with incidences of high blood pressure, high cholesterol, and diabetes in model.

**Table 4:** Estimated Hazard Ratios (95% CI) for self-reported morbidities in 2005–2013 for an increment of IQR increase in pollutant concentrations.

	PM_10_ (μg/m^3^)	O_3_ (ppb)	NO_2_ (ppb)	SO_2_ (ppb)	CO (ppm)
**High blood pressure**					
Model I	1.52 (1.40, 1.64)*	0.82 (0.74, 0.91)*	0.95 (0.88, 1.03)	1.93 (1.73, 2.14)*	1.15 (1.08, 1.24)*
Model II	1.55 (1.43, 1.67)*	0.77 (0.70, 0.86)*	0.94 (0.86, 1.01)	2.28 (2.05, 2.54)*	1.16 (1.08, 1.24)*
Model III	1.54 (1.42, 1.67)*	0.77 (0.69, 0.86)*	0.93 (0.86, 1.02)	2.32 (2.07, 2.60)*	1.15 (1.07, 1.24)*
Model IV	1.13 (1.04, 1.23)*	0.89 (0.80, 0.99)*	1.01 (0.94, 1.09)	1.22 (1.08, 1.38)*	1.07 (1.00, 1.15)
Model V	1.18 (1.07, 1.30)*	0.90 (0.80, 1.02)	1.03 (0.94, 1.13)	1.66 (1.34, 2.07)*	1.09 (1.00, 1.18)*
**High cholesterol**					
Model I	1.62 (1.54, 1.69)*	0.83 (0.78, 0.89)*	0.89 (0.85, 0.94)*	1.82 (1.71, 1.95)*	1.03 (0.99, 1.08)
Model II	1.62 (1.55, 1.70)*	0.80 (0.75, 0.86)*	0.87 (0.82, 0.92)*	2.06 (1.92, 2.20)*	1.02 (0.97, 1.07)
Model III	1.63 (1.55, 1.70)*	0.80 (0.75, 0.86)*	0.85 (0.81, 0.90)*	2.11 (1.96, 2.26)*	1.02 (0.97, 1.07)
Model IV	1.07 (1.02, 1.12)*	1.00 (0.93, 1.07)	0.97 (0.92, 1.02)	1.20 (1.11, 1.30)*	1.02 (0.97, 1.07)
Model V	1.10 (1.04, 1.16)*	1.03 (0.95, 1.11)	0.93 (0.87, 0.99)*	1.68 (1.48, 1.91)*	1.02 (0.96, 1.07)
**Diabetes**					
Model I	1.71 (1.48, 1.96)*	0.83 (0.68, 1.01)	0.95 (0.83, 1.10)	1.71 (1.40, 2.10)*	1.07 (0.93, 1.22)
Model II	1.76 (1.53, 2.02)*	0.78 (0.64, 0.95)*	0.92 (0.79, 1.07)	2.15 (1.74, 2.65)*	1.05 (0.92, 1.21)
Model III	1.81 (1.56, 2.09)*	0.81 (0.66, 1.00)*	0.89 (0.75, 1.05)	2.19 (1.75, 2.75)*	1.04 (0.89, 1.20)
Model IV	1.05 (0.91, 1.21)	0.97 (0.78, 1.21)	1.04 (0.89, 1.22)	1.21 (0.92, 1.60)	1.06 (0.92, 1.22)
Model V	1.09 (0.91, 1.30)	1.01 (0.79, 1.30)	0.97 (0.81, 1.17)	1.88 (1.24, 2.85)*	1.00 (0.85, 1.18)

Significance indicated by *P < 0.05. Model I: no adjustment; Model II: adjusted for age and sex; Model III: further adjusted for BMI, smoking status, alcohol intake, physical activity, food consumption, intake of fruit and vegetables, marital status, education level, and income. Specifically, food consumption in model III: high blood pressure models adjusted for high sodium consumption; high cholesterol models adjusted for high fat consumption; diabetes models adjusted for high sugar consumption and sugar-sweetened soft drink consumption. Model IV (Main model): further adjusted for temperature and relative humidity; Model V: further adjusted for other self-reported comorbidities and city of residence.

Although the HRs of high blood pressure and cholesterol exceeded 1.5 per IQR increase in PM_10_ and SO_2_ in Model I, II and III, the associations became weaker in Model IV (main model) which adjusted for 2-year average of temperature and relative humidity. In Model IV, the HRs of PM_10_ were 1.13 (95%CI: 1.04, 1.23) for high blood pressure and 1.07 (95%CI: 1.02, 1.12) for high cholesterol. The HRs of SO_2_ were 1.22 (95%CI: 1.08, 1.38) for high blood pressure and 1.20 (95%CI: 1.11, 1.30) for high cholesterol, which were slightly higher than those of PM_10_. Although the association of SO_2_ with diabetes was not significant in the main model (HR = 1.21, 95%CI: 0.92, 1.6), the association became significant (HR = 1.88, 95%CI: 1.24, 2.85) after adjusted for other self-reported co-morbidities and city of residence (Model V). We found no clear association between PM_10_ with incidences of diabetes (HR = 1.05, 95%CI: 0.91, 1.21), and all self-reported morbidities with O_3_ and NO_2_ in the main model and our sensitivity analysis in Model V. In two-pollutants model (Table S1), the associations of PM_10_ and SO_2_ with incidences of self-morbidities were not essentially changed after adjusted by co-pollutants.

## 4 Discussions

In this study, we examined the association of long-term air pollution with self-reported morbidity of high blood pressure, high cholesterol, and diabetes in subjects of TCS in BMR, Thailand. Long-term exposure to PM_10_ was positively associated with incidences of high blood pressure and high blood cholesterol, but not for diabetes in the main model. Additionally, we also found the positive association of SO_2_ with all self-reported morbidities, and CO with incidence of high blood pressure. However, we did not observe the clear association of O_3_ and NO_2_ with self-reported morbidities.

Recent longitudinal studies have consistently reported that an increase of air pollutant concentrations (e.g. PM_2.5_, NO_2_, NOx) were associated with hypertension incidence (Bai et al., 2018; Bo et al., 2019b; Coogan et al., 2012), diabetes morbidity (Andersen et al., 2012; Bai et al., 2018; Coogan et al., 2012; Lao et al., 2019; Liang et al., 2019; Qiu et al., 2018) and mortality (Brook et al., 2013; Lim et al., 2018). Nonetheless, some previous studies did not find the association of long-term exposure to air pollution with hypertension and diabetes (Adar et al., 2018; Chen et al., 2015; Lazarevic et al., 2015). In this study, we observed significant positive associations of hypertension with PM_10_ and CO, but not with O_3_ and NO_2_. Long-term SO_2_ exposure was also positively associated with hypertension and diabetes. However, we did not find the clear association between diabetes with other pollutants in the main model.

Several biological mechanisms linked particulate air pollution to the development of hypertension and diabetes, include the elicitation of local and systemic inflammation and oxidative stress, endothelial dysfunction, and the triggering of autonomic nervous system imbalance (Brook et al., 2004, 2009, 2010; Brook and Rajagopalan, 2009; Coogan et al., 2012). Other proposed mechanisms connecting air pollution exposure and promotion of insulin resistance have also been suggested by animal and human studies (Evans et al., 2002; Kelly, 2003; Liu et al., 2014; Rajagopalan and Brook, 2012; Sun et al., 2009). In addition, the trigger of autonomic nervous system imbalance by particulate matter which can promote to vasoconstriction (Brook, 2008; Brook et al., 2002), contributes to hypertension and impaired insulin sensitivity (Carnethon et al., 2003; Sun et al., 2009).

Previous studies observed the associations between long-term PM_2.5_ exposure with dyslipidemias incidence (Bo et al., 2019a) and prevalence (Mao et al., 2020). Similarly, we also found the positive association between PM_10_ and SO_2_ with incident high blood cholesterol, but not for other pollutants. The biological mechanisms underlying the relationship between long-term exposure to air pollution and changed blood lipids is still unclear. Some evidences suggested that air pollution inhalation could induce inflammation and oxidative stress, interfering with lipids metabolism and oxidation, and contributing to altered blood lipid levels (Araujo and Nel, 2009; Mao et al., 2020; Poursafa et al., 2014; Xu et al., 2011). Besides, intervention (Chen et al., 2016) and experimental (Mendez et al., 2013) studies suggested that decreases in DNA methylation (Bind et al., 2014), especially on genes elicited by inhaled air pollution also related to lipid metabolism and inflammation pathways.

In this study, Cox regression model was selected in order to compare our results of HRs to previous studies which also used the same method to examine the associations between long-term exposure to air pollutants, especially for PM_10_ and PM_2.5_ and the incidences of dyslipidemia (Bo et al., 2019a), diabetes and hypertension (Andersen et al., 2012; Bai et al., 2018; Coogan et al., 2012; Lao et al., 2019; Liang et al., 2019; Qiu et al., 2018). The magnitude of the effects for PM_10_ in our main model (model IV), which is represented by HRs, is not much different to most of the previous studies. For example, Yang et al. (2020) showed meta-analyses of diabetes incidence with PM_2.5_ (11 studies; HR = 1.10, 95% CI = 1.04–1.17 per 10 μg/m^3^ increment) and PM_10_ (6 studies; HR = 1.11; 95% CI = 1.00–1.22 per 10 μg/m^3^ increment). However, few previous studies observed the magnitude of HRs between PM_2.5_ and the incidences of hypertension and diabetes (Coogan et al., 2012; Lao et al., 2019) larger than our study. The evidences of long-term effects of SO_2_ on morbidities, especially for the incidences of diabetes and high blood cholesterol are still limited. Although the sample size of our study was smaller than most of previous studies, data richness in terms of detailed availability of potential confounding allows a thorough adjustment in the model resulting to a more robust estimation.

Many previous cross-sectional studies might not be able to detect the association between air pollution and hypertension, high cholesterol, and diabetes because these diseases are a chronic process (Chen et al., 2015; Lazarevic et al., 2015). Furthermore, air pollution exposure in some studies (Chuang et al., 2011; Shanley et al., 2016) were estimated based on the proximity of residences to fixed monitoring stations, assigning the same exposure level to entire communities (districts, counties or cities) which may introduce misclassification and inconsistent results. In this longitudinal study, we are able to detect developments or changes in the characteristics of the target population at both the group and the individual level because several observations of the same subjects were conducted over a period of time. Additionally, we also used ordinary kriging method to evaluate spatial representativeness of monitoring stations which can improve the accuracy of air pollution exposure estimates.

Previous studies from TCS suggested the characteristic factors which have been reported to correlate with morbidities of high blood pressure, high blood lipids, and diabetes (Papier et al., 2016, 2017; Rimpeekool et al., 2017; Seubsman et al., 2010). Rimpeekool et al. (2017) reported increased risk of high blood pressure, high blood lipids, and obesity among Thai adults who have low physical activity, unhealthy eating (high levels of sugar, fat and sodium, and little fibre), and seldom/rarely use nutritional labelling. In men and women, type 2 diabetes mellitus (T2DM) was positively associated with age, BMI, smoking, and alcohol intake (Papier et al., 2016). The sociodemographic (i.e. education, income, household assets, and housing type) and lifestyle changes that have been accompanied with Thailand's economic development were also associated strongly with obesity and T2DM risk in a large cohort of Thai adults (Papier et al., 2016; Seubsman et al., 2010). Seubsman et al. (2010) reported that obesity increased with age and was more prevalent among males than females. In addition, Thais who lived in urban residence associated with unhealthy diets for both sexes (Papier et al., 2017).

Our study has several potential limitations. First, we can include only subjects who lived in BMR from 2005 until 2013 due to the availability of air pollution data. Moreover, the study period was also based on the TCS which started in 2005 and ended in 2013, where this 9-year period is long enough, to some extent, to observe long-term health effects of air pollution in the study area. Second, we could not include traffic variables and land use data into the ordinary kriging due to the limitations of the data in Thailand. However, we used air pollution data from traffic monitoring stations for exposure assessment to generate ordinary kriging for traffic-related air pollution such as NO_2_ and CO. Third, self-report of disease diagnosis was also a limitation. Although the diseases that each subject got in this study were diagnosed and confirmed by the doctor, we knew only the year that each subject got disease but we did not know the date. Hence, we can only match the health outcomes data with annual air pollution exposure.

Notwithstanding, most of the cohort members were relatively young (aged 20–39 years old) and had not developed cardiovascular disease yet, we may find the association between air pollution and cardiovascular disease risk factors for younger population from this study. Thus, this study may clarify the process from exposure to air pollutants to development of cardiovascular diseases, by affecting risk factors. A lot of data was also recorded in this study such as BMI, education level, income, marital status, regular exercise, alcohol consumption, smoking status, food and drink consumption, intake of vegetables and fruit. Therefore, we can control these variables in our model. Furthermore, we also applied ordinary kriging method which can evaluate spatial representativeness of monitoring stations and improve the accuracy of air pollution exposure estimates.

## 5 Conclusions

Long-term exposure to air pollution, particularly for PM_10_ and SO_2_ was associated with self-reported high blood pressure, high blood cholesterol, and diabetes in subjects of TCS. Our findings could be a benefit and helpful for understanding long-term effects of air pollution on risk factors for cardiovascular diseases, as well as their mechanisms under current situation in Thailand. Moreover, this study may contribute to the establishment and improvement of long-term air pollution control strategies in Thailand for preventing public health issues. Further epidemiological studies are needed to understand and identify plausible mechanisms underlying the association, as well as longitudinal studies to confirm the causal relationship between long-term air pollution exposure with diabetes and high blood cholesterol. In the advent of more granular exposure data, further studies can focus on the sources of air pollution and develop new or advanced method for exposure assessment, as well as long-term effects of weather on the morbidities.

## CRediT author statement

**Kanawat Paoin**: Conceptualization, Formal analysis, Writing - original draft. **Kayo Ueda**: Conceptualization, Supervision, Writing - review & editing. **Thammasin Ingviya**: Methodology, Writing - review & editing. **Suhaimee Buya**: Methodology. **Arthit Phosri**: Methodology, Writing - review & editing. **Xerxes Tesoro Seposo**: Methodology, Writing - review & editing. **Sam-ang Seubsman**: Data collection, Data curation, Funding acquisition. **Matthew Kelly**: Supervision, Writing - review & editing. **Adrian Sleigh**: Supervision, Writing - review & editing, Funding acquisition. **Akiko Honda**: Supervision. **Hirohisa Takano**: Supervision.

## Ethics and consent

Approval for the study was obtained from Sukhothai Thammathirat Open University Research and Development Institute (protocol number 0522/10) and the Australian National University Human Research Ethics Committee (protocol numbers 2004/344 and 2009/570) and the Graduate School of Engineering, Kyoto University.

## Declaration of competing interest

The authors declare that they have no known competing financial interests or personal relationships that could have appeared to influence the work reported in this paper.
