# Supplementary material for: Long-term air pollution exposure and self-reported morbidity: A longitudinal analysis from the Thai cohort study (TCS)
Source: Environ Res. 2021 Jan;192:110330. doi: 10.1016/j.envres.2020.110330 (PMC7768181; doi:10.1016/j.envres.2020.110330)
Supplement: Multimedia component 1 [file mmc1.docx]

Table S1. Estimated Hazard Ratios (95% CI) for self-morbidities in 2005-2013

for air pollution levels in two-pollutant model

|  | **High blood pressure** | **High blood cholesterol** | **Diabetes** |
| --- | --- | --- | --- |
| **PM_10_** |  |  |  |
| + O_3_ | 1.11 (1.01, 1.22)* | 1.09 (1.03, 1.14)* | 1.05 (0.89, 1.24) |
| + NO_2_ | 1.15 (1.05, 1.26)* | 1.09 (1.04, 1.14)* | 1.04 (0.89, 1.21) |
| + SO_2_ | 1.16 (1.07, 1.26)* | 1.09 (1.04, 1.14)* | 1.07 (0.92, 1.23) |
| + CO | 1.12 (1.03, 1.22)* | 1.07 (1.02, 1.12)* | 1.05 (0.91, 1.21) |
| **SO_2_** |  |  |  |
| + PM_10_ | 1.28 (1.13, 1.45)* | 1.23 (1.14, 1.33)* | 1.24 (0.93, 1.64) |
| + O_3_ | 1.20 (1.06, 1.36)* | 1.21 (1.12, 1.31)* | 1.21 (0.91, 1.61) |
| + NO_2_ | 1.25 (1.10, 1.42)* | 1.20 (1.11, 1.30)* | 1.26 (0.94, 1.69) |
| + CO | 1.23 (1.08, 1.39)* | 1.20 (1.11, 1.30)* | 1.22 (0.92, 1.61) |

All models were 4th model. Significance indicated by: * P-value < 0.05
